# Anatomy of the maxillary canal of *Riograndia guaibensis* (Cynodontia, Probainognathia)—A prozostrodont from the Late Triassic of southern Brazil

**DOI:** 10.1002/ar.25540

**Published:** 2024-07-22

**Authors:** Pedro Henrique Morais Fonseca, Agustín Guillermo Martinelli, Pamela G. Gill, Emily J. Rayfield, Cesar Leandro Schultz, Leonardo Kerber, Ana Maria Ribeiro, Marina Bento Soares

**Affiliations:** ^1^ Programa de Pós‐Graduação em Geociências, Instituto de Geociências Universidade Federal do Rio Grande do Sul Porto Alegre Brazil; ^2^ CONICET‐Sección Paleontología de Vertebrados Museo Argentino de Ciencias Naturales “Bernardino Rivadavia” Buenos Aires Argentina; ^3^ Núcleo Milenio EVOTEM‐Evolutionary Transitions of Early Mammals‐ANID Santiago Chile; ^4^ Palaeobiology Research Group, School of Earth Sciences University of Bristol, Life Sciences Building Bristol UK; ^5^ Earth Sciences Department The Natural History Museum London UK; ^6^ Centro de Apoio à Pesquisa Paleontológica Universidade Federal de Santa Maria São João do Polêsine Brazil; ^7^ Seção de Paleontologia, Museu de Ciências Naturais, Secretaria do Meio Ambiente e Infraestrutura do Rio Grande do Sul Porto Alegre Brazil; ^8^ Departamento de Geologia e Paleontologia, Museu Nacional Universidade Federal do Rio de Janeiro Rio de Janeiro Brazil

**Keywords:** anatomy, Brazil, computed tomography, South America, Triassic, trigeminal nerve

## Abstract

Investigating the evolutionary trajectory of synapsid sensory and cephalic systems is pivotal for understanding the emergence and diversification of mammals. Recent studies using CT‐scanning to analyze the rostral foramina and maxillary canals morphology in fossilized specimens of probainognathian cynodonts have contributed to clarifying the homology and paleobiological interpretations of these structures. In the present work, μCT‐scannings of three specimens of *Riograndia guaibensis*, an early Norian cynodont from southern Brazil, were analyzed and revealed an incomplete separation between the lacrimal and maxillary canals, with points of contact via non‐ossified areas. While the maxillary canal exhibits a consistent morphological pattern with other Prozostrodontia, featuring three main branches along the lateral region of the snout, the rostral alveolar canal in *Riograndia* displays variability in the number of extra branches terminating in foramina on the lateral surface of the maxilla, showing differences among individuals and within the same skull. Additionally, pneumatization is observed in the anterior region of the skull, resembling similar structures found in reptiles and mammals. Through this pneumatization, certain branches originating from the maxillary canal extend to the canine alveolus. Further investigation is warranted to elucidate the functionality of this structure and its occurrence in other cynodont groups.

## 1 INTRODUCTION

Investigating the evolutionary trajectory of the synapsid sensory and cephalic systems is of significant relevance to understanding the emergence and diversification of mammals (Benoit, Fernandez, et al., 2016; Benoit, Manger, et al., 2016; Fonseca et al., 2024; Heesy & Hall, 2010; Kerber, Roese‐Miron, et al., 2024; Moore, 1981; Norton et al., 2023; Rowe & Shepherd, 2016; Rowe et al., 2011; Rowe, 2020). Some of the morphological changes that occurred during the evolution of probainognathians during the Late Triassic are possibly related to nocturnality, such as the development of a more refined olfactory system and the development of sensory whiskers (Benoit, Fernandez, et al., 2016; Benoit, Manger, et al., 2016; Heesy & Hall, 2010; Rowe, 2020). Concerning this latter aspect, since the beginning of the 20th century, the origin of sensory whiskers in synapsids has been studied and debated by several authors (e.g. Benoit et al., 2020; Benoit, Fernandez, et al., 2016; Benoit, Manger, et al., 2016; Kemp, 1983; Pocock, 1914; Watson, 1931). As nerves and soft tissues are rarely preserved in fossils, paleontologists analyze the rostral foramina and the morphology of the maxillary canal to propose and discuss paleobiological interpretations.

In modern mammals, concerning the rostral foramina relevant to this study, only one opening, namely the infraorbital foramen, is responsible for the exit of the branch of the trigeminal nerve that innervates the whiskers (Schaller, 1992). However, the history of this foramen is complex, particularly in non‐mammaliaform cynodonts and early mammaliaforms. These taxa exhibit a greater number of foramina (Benoit et al., 2020; Benoit, Fernandez, et al., 2016; Benoit, Manger, et al., 2016), which makes it challenging to ascertain the homology of such structures with the mammalian infraorbital foramen. Until recently, most studies that considered the exits of the maxillary ramus of the trigeminal nerve were based on the topology of such foramina along the snout (e.g. Bonaparte et al., 2005; Hopson & Kitching, 2001; Kemp, 1983; Kermack et al., 1981; Martinelli et al., 2005; Oliveira et al., 2011; Stefanello et al., 2023; Sues, 1986).

The maxillary canal, located in the rostral portion of the cranium, has been studied in synapsids and in several other tetrapod groups. This feature is one of the most important sources of information on facial innervation in extinct species (e.g. Fourie, 1974; Kemp, 1983; Kermack et al., 1981; Kühne, 1956), providing evidence on the sensory function of the snout (e.g. Asahara et al., 2016; Barker et al., 2017; Benoit et al., 2018, 2020; Benoit, Fernandez, et al., 2016; Benoit, Manger, et al., 2017; Benoit, Manger, et al., 2016; Benoit, Norton, et al., 2017; Crompton et al., 2015, 2017; Dal Sasso et al., 2005; Ibrahim et al., 2014; Kermack et al., 1981; Laaß & Kaestner, 2017; Leitch & Catania, 2012). In the case of synapsids, the evolution of the maxillary canal is intricately linked to the presence of whiskers. Analyzing the changes in the branches of this canal allows for inferences on the potential presence or absence of whiskers, as well as interpretations of the origin of mammalian traits (Benoit et al., 2020; Benoit, Fernandez, et al., 2016; Benoit, Manger, et al., 2016). In most non‐mammaliaform cynodonts, the maxillary canal has a longer path inside the bone, and more exits for branches, demonstrating that this structure underwent major changes throughout the evolutionary history of the group (e.g. Benoit et al., 2020).

Recently, with the potential of CT‐scanning to access endocranial morphology, the foramina of the snout and maxillary canal, as well as the path of innervation of the trigeminal nerve, have been addressed in several non‐mammaliaform cynodonts, facilitating a comprehensive understanding of the homology and function of such openings (e.g. Benoit, Fernandez, et al., 2016; Benoit, Manger, et al., 2016; Benoit et al., 2020; Franco et al., 2021; Kerber, Roese‐Miron, et al., 2024; Wallace et al., 2019). This includes several probainognathians of major interest in understanding the origin of mammalian traits. Although a relatively large sample of non‐mammaliaform cynodonts has been studied so far, detailed descriptions for key taxa and inter‐ and intraspecific variations on this issue are still pending.

*Riograndia guaibensis* is a prozostrodont probainognathian from the early Norian *Riograndia* Assemblage Zone (Candelária Sequence, Santa Maria Supersequence) in southern Brazil (Bonaparte et al., 2001, 2010; Soares et al., 2011). It is an important taxon because it has a unique combination of traits, such as enlarged incisors, leaf‐shaped postcanine teeth, short and tall snout, robust forelimbs that make this taxon unique among probainognathians (Bonaparte et al., 2001; Fonseca et al., 2024; Guignard et al., 2019; Soares et al., 2011). Furthermore, it has the potential to yield important new neurosensory information within the lineage that gave rise to mammaliaforms (Liu & Olsen, 2010). In this study, we present an anatomical description and document intraspecific variations of the maxillary canal based on three specimens of this cynodont.

## 2 MATERIALS AND METHODS

### 2.1 Specimens and provenance

**FIGURE 1:**
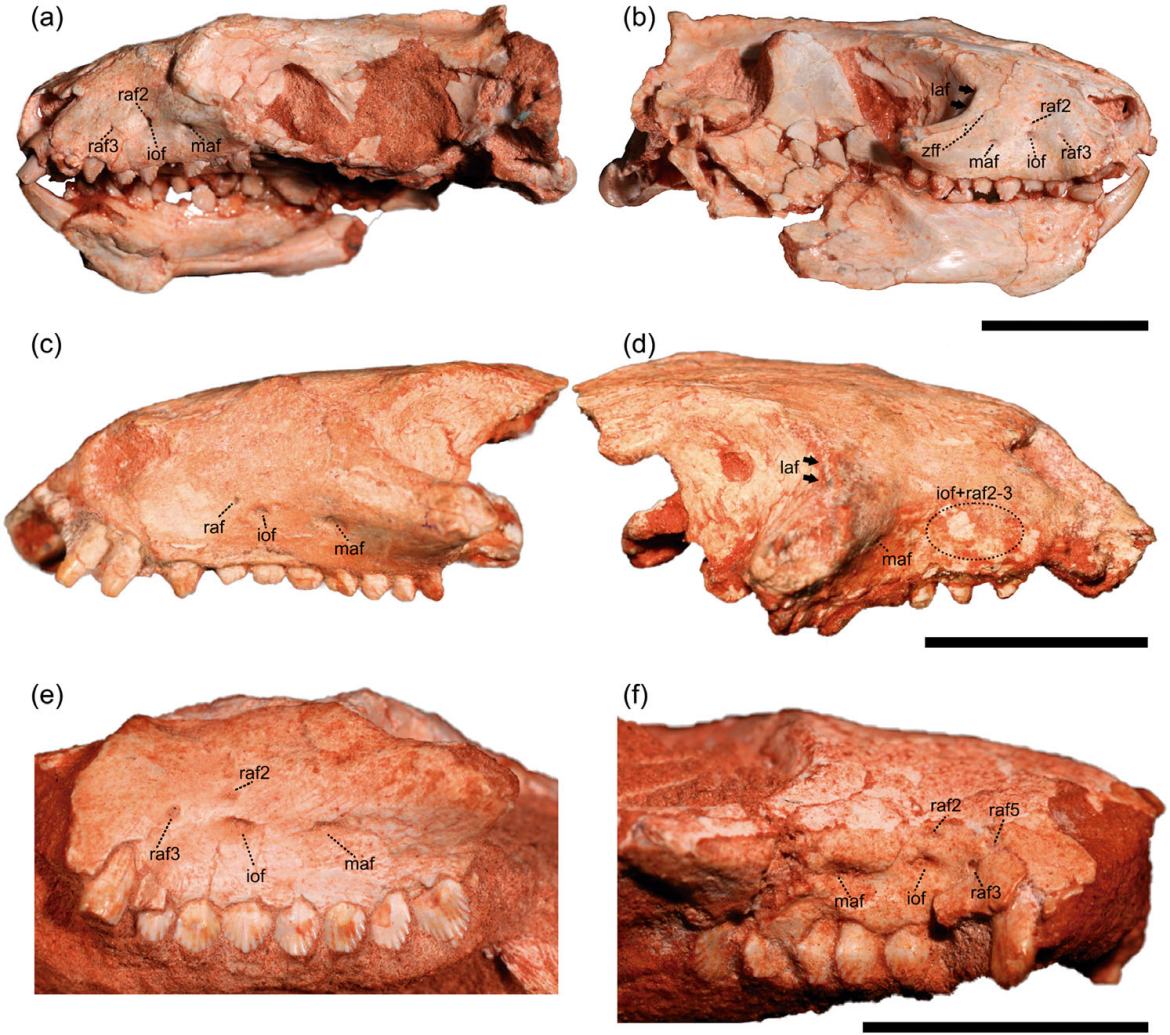
Studied specimens of *Riograndia guaibensis*. UFRGS‐PV‐596‐T in left (a) and right (b) lateral views; holotype MCN‐PV 2264 in left (c) and right (d) lateral views; UFRGS‐PV‐788‐T in left (e) and right (f) lateral view. iof, infraorbital foramen; laf, lacrimal foramen; maf, middle alveolar foramen; raf, rostral alveolar foramen; raf2, rostral alveolar foramen, second branch; raf3, rostral alveolar foramen, third branch; raf5, rostral alveolar foramen, fifth branch; zff, zygomaticofacial foramen. Scale bars: 1 cm.

The studied material includes the holotype MCN‐PV 2264 of *Riograndia guaibensis* and the referred specimens UFRGS‐PV‐596‐T and UFRGS‐PV‐788‐T (Figure 1). They were published in Bonaparte et al. (2001) and Soares et al. (2011). MCN‐PV 2264 comes from Sesmaria do Pinhal 1 outcrop, Candelária municipality, state of Rio Grande do Sul (Brazil), and UFRGS‐PV‐596‐T and UFRGS‐PV‐788‐T come from Linha São Luiz outcrop, Faxinal do Soturno municipality, State of Rio Grande do Sul (Brazil). Both localities expose outcrops that are referred to the *Riograndia* Assemblage Zone, early Norian of the Candelária Sequence, Santa Maria Supersequence (Martinelli et al., 2021; Schultz et al., 2020).

### 2.2 Scanning procedures and three‐dimensional modeling

The specimens UFRGS‐PV‐596‐T and UFRGS‐PV‐788‐T were scanned with a μCT NIKON XTH225ST, at the School of Earth Sciences, University of Bristol, United Kingdom. Specimen UFRGS‐PV‐596‐T was scanned at 180 kV and 38 μA with a voxel size of 0.012324 mm, and the specimen UFRGS‐PV‐788‐T was scanned at 155 kV and 171 μA with a voxel size of 0.02449 mm. Specimen MCN‐PV 2264 was scanned with a Bruker SkyScan 1173 at the Instituto de Petróleo e dos Recursos Naturais (Laboratório de Sedimentologia e Petrologia) of Pontifícia Universidade Católica do Rio Grande do Sul, Porto Alegre, Brazil. MCN‐PV 2264 was scanned at 80 kV and 100 μA with a voxel size of 0.01411 mm. All tomographic images were edited and analyzed in Avizo (version 7.1, 8.1, and 9.1, Visualization Sciences Group), through manual editing using a tablet One by WACOM.

### 2.3 Anatomical nomenclature

The nomenclature of the structures and the name of the branches of the maxillary canal described here are based on Benoit, Fernandez, et al. (2016), Benoit, Manger, et al. (2016), and Benoit et al. (2020).

### 2.4 Phylogenetic analysis

To contribute to knowledge of the anatomy and phylogenetic relationships of *Riograndia guaibensis*, we scored the maxillary canal and recess characters (characters 150–154) proposed by Benoit et al. (2022) in an updated Eucynodontia matrix. The matrix is based on Liu and Olsen (2010) and it was modified over the years by several authors (e.g. Kerber, Pretto, et al., 2024; Kerber, Roese‐Miron, et al., 2024; Martinelli, Eltink, et al., 2017; Martinelli, Soares, et al., 2017; Soares et al., 2014; Wallace et al., 2019; see Martinelli et al., 2024 for details). The data matrix includes 158 characters and 45 operational taxonomic units. The analysis was processed in the software TNT v.1.5 (Goloboff & Catalano, 2016). The early diverging cynodont *Procynosuchus delaharpeae* was used to root the most parsimonious trees, which were recovered with a heuristic search (random addition sequence + tree bisection reconnection), with 1000 replicates of Wagner trees (with random seed = 0) and using tree bisection reconnection and branch swapping (holding 10 trees save per replication). All characters received the same weight and were treated as unordered (i.e., non‐additive). The data matrix is available as Supplementary Information in Nexus and TNT files.

### 2.5 Institutional abbreviations

UFRGS, Universidade Federal do Rio Grande do Sul, Porto Alegre, Rio Grande do Sul, Brazil; MCN, Museu de Ciências Naturais, Secretaria Estadual do Meio Ambiente e Infraestrutura, Porto Alegre, Rio Grande do Sul, Brazil.

## 3 RESULTS

### 3.1 Description and comparisons

*Riograndia guaibensis* is characterized by a short, tall, and wide rostrum, with a unique dentition, including three procumbent incisors, a reduced canine, and up to eight–nine transversely narrow postcanine teeth with leaf‐shaped crowns (Bonaparte et al., 2001; Soares et al., 2011). The preorbital region represents ca. 33% of the skull basal length, according to the measurements taken from UFRGS‐PV‐596‐T and UFRGS‐PV‐788‐T.

**FIGURE 2:**
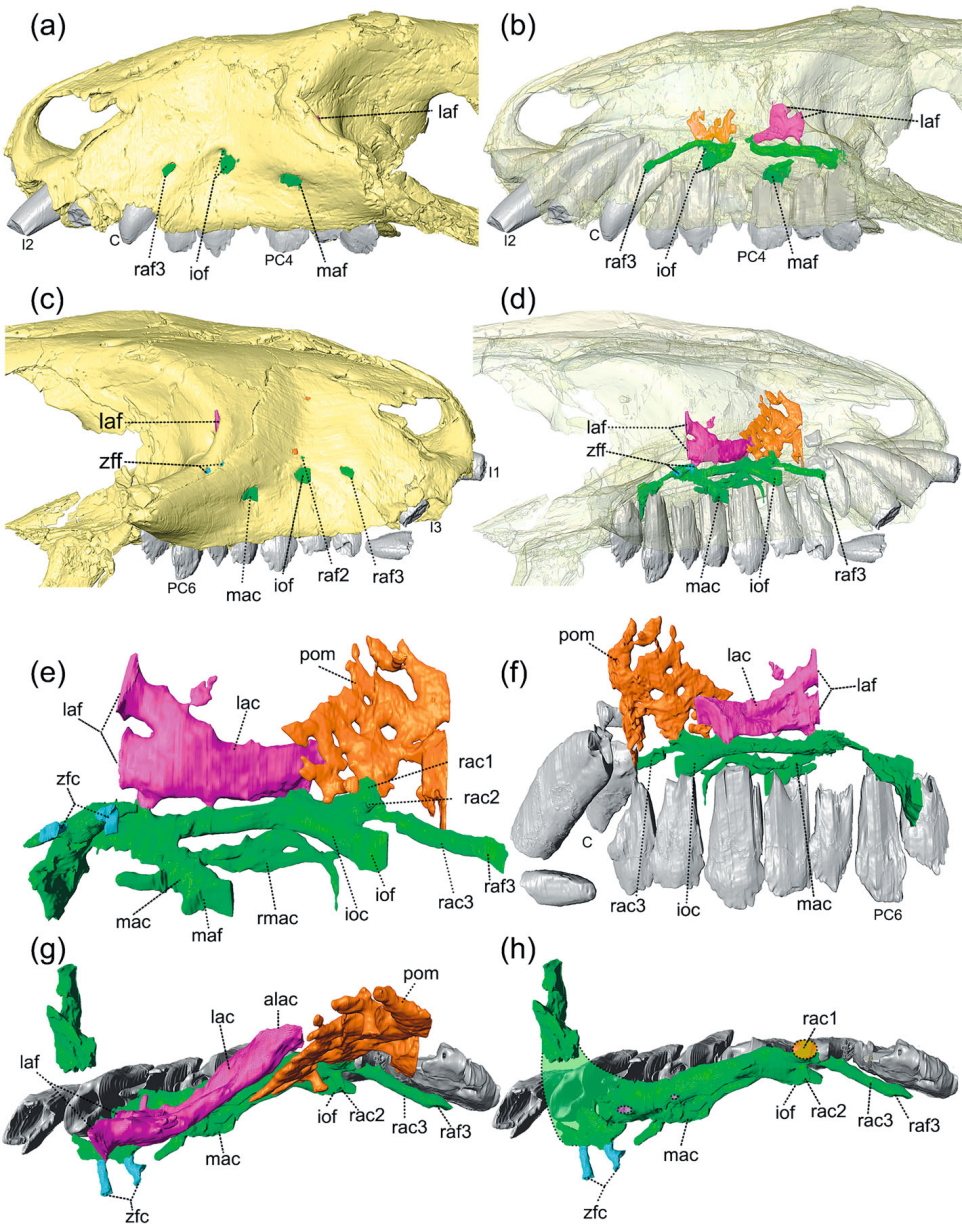
Three‐dimensional models of the rostral region of *Riograndia guaibensis* UFRGS‐PV‐596‐T and the digital filling of the maxillary canal (in green), lacrimal canal (in pink), and pneumatization of the maxilla (in orange). The left side of the snout in lateral view in (a) and (b) and the right side in lateral view in (c) and (d). In (b) and (d) the bones are translucent. In (e) the filled canals are in lateral and (f) in medial views. In (g) and (h) the filled canals are in dorsal view. In (h) the lacrimal canal (in pink) and pneumatization of the maxilla (in orange) were removed. In (h) the green dotted line represents the reconstruction of the posterior region of the maxillary canal and the dotted line filled with orange the contact of the maxillary canal with the pneumatization of the maxilla. alac, anterior opening of the lacrimal canal; C, upper canine; I1, first upper incisor; I2, second upper incisor; I3, third upper incisor; ioc, infraorbital canal; iof, infraorbital foramen; lac, lacrimal canal; laf, lacrimal foramen; mac, middle alveolar canal; maf, middle alveolar foramen; PC4, fourth upper postcanine; PC6, sixth upper postcanine; pom, pneumatization of the maxilla; rac1, rostral alveolar canal, first branch; rac2, rostral alveolar canal, second branch; rac3, rostral alveolar canal, third branch; raf3, rostral alveolar foramen, third branch; zfc, zygomaticofacial canal; zff, zygomaticofacial foramen.

Figure 1 shows the exits of the trigeminal nerve recognized in the studied specimens, on both lateral sides of the rostrum. Based on the CT reconstruction, the lacrimal canal runs dorsally to the maxillary canal, inside the lacrimal and maxillary bone (Figure 2). Posteriorly, the lacrimal canal opens into two foramina inside the orbit in the lacrimal bone and anteriorly opens inside the nasal cavity at the contact between the maxillary and lacrimal bone. The lacrimal canal is morphologically similar to *Brasilodon quadrangularis* (Benoit et al., 2020), being shorter than the maxillary canal. However, the lacrimal canal of *Riograndia* is comparatively shorter than in *Brasilodon*, due to the shortening of the snout of the former taxon. Benoit et al. (2020) noted that the maxillary canal completely ossified and separated from the lacrimal canal is the derived condition within non‐mammaliaform cynodonts, according to their description of *B. quadrangularis*, *Tritylodon longaevus*, and *Pachygenelus monus*. Nonetheless, the lacrimal canal in *Riograndia* presents at least two intersection points with the maxillary canal (Figures 2h and 5). The most posterior intersection is at the level of the fifth postcanine, where it forms a small canal and has a medium diameter in comparison to the other opening. The second intersection is the smallest in diameter and also forms a canal. The points of contact between the lacrimal and maxillary canals were not mentioned for other taxa in Benoit et al. (2020).

The general morphology of the maxillary canal of *Riograndia* (UFRGS‐PV‐596‐T, UFRGS‐PV‐788‐T, MCN‐PV 2264) is similar to the condition present in *Brasilodon* and other Prozostrodontia, such as *Tritylodon* and *Pachygenelus* (Benoit et al., 2020). The maxillary canal in *Riograndia* begins at the interorbital wall, through the maxillary foramen (sensu Benoit et al., 2020; Krause et al., 2014), into the lacrimal bone, following the anterior margin of the lacrimal, and reaching the maxillary bone. Inside the maxilla, the maxillary canal runs dorsolaterally to the roots of the tooth row (Figure 2). In dorsal view, the maxillary canal runs laterally in relation to the tooth row, for almost its entire length, except for its posterior most portion, which bends medially following the anterior margin of the lacrimal at the level of the fifth postcanine, projecting up to the interorbital wall. The anterior portion of the maxillary canal is divided into three branches, as in prozostrodonts (Benoit et al., 2020; Kerber, Roese‐Miron, et al., 2024). The lengthening of the rostral alveolar canal compared to the infraorbital canal in *Riograndia* matches the pattern expected for prozostrodonts (Benoit et al., 2020). Furthermore, the maxillary canal contacts the pneumatization of the maxilla, which presents a canal‐shaped opening at the level of the base of the rostral alveolar canal. This structure comes into contact with the maxillary canal and alveoli of the canine and first postcanine, through which the nerves and vessels that supply the teeth should pass.

The most posterior branch of the maxillary canal is the middle alveolar canal which branches at the level of the fourth postcanine and ends in a single foramen in the maxilla (Figure 1). It is longer than the infraorbital canal and smaller than the rostral alveolar canal but has a similar diameter in cross section to that of the infraorbital canal. In the specimen UFRGS‐PV‐596‐T, the middle alveolar canal has a projection on the right side of the skull, only observed in this individual. The projection includes two branches, one posterior and one anterior to the middle alveolar canal. The posterior branch is shorter than the anterior one and ends inside the anterior portion of the zygomatic arch. The anterior branch is longer than the posterior one and is divided into two canals. One of the canals is straight and the other has a dorsal curvature that projects anteriorly and culminates into the alveolus of the second postcanine tooth. In *Riograndia*, the opening is on the lateral surface of the maxilla, on the anterior border of the zygomatic arch which differs from *Brasilodon*, *Tritylodon*, and *Pachygenelus*, where the opening of the middle alveolar canal faces ventral to the base of the zygomatic arch in the maxilla (Benoit et al., 2020).

**FIGURE 3:**
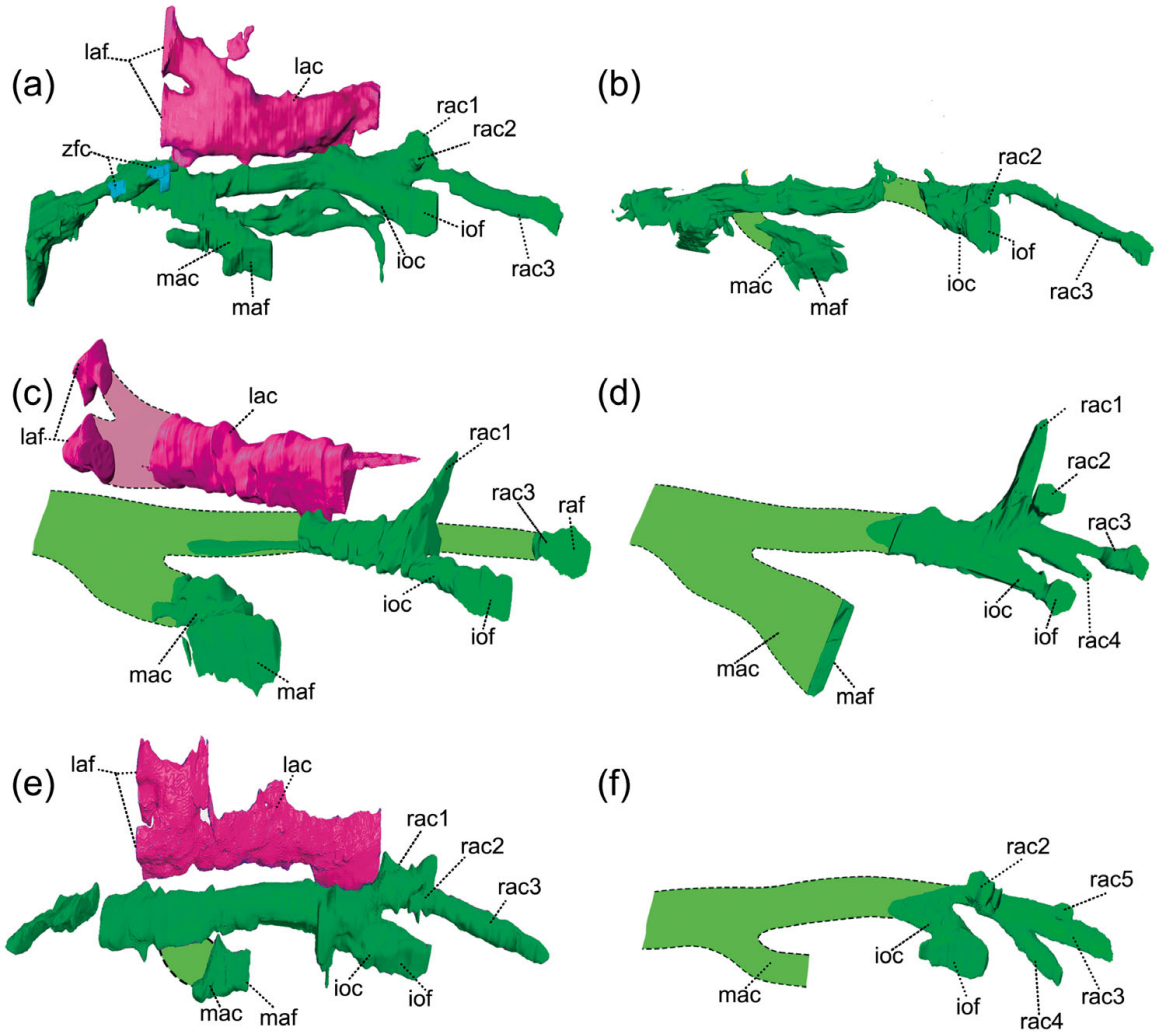
Three‐dimensional models of the filling of the maxillary canal (in green) and lacrimal canal (in pink) in different specimens of *Riograndia guaibensis*. The dashed lines represent the reconstruction of the parts of the maxillary canal that were not possible to be recovered using tomography. UFRGS‐PV‐596‐T, right maxillary canal (a) and mirrored left maxillary canal (b); holotype MCN‐PV 2264, mirrored left maxillary canal (c) and right maxillary canal (d); UFRGS‐PV‐788‐T, mirrored left maxillary canal (e) and right maxillary canal (f). ioc, infraorbital canal; iof, infraorbital foramina; lac, lacrimal canal; laf, lacrimal foramen; mac, middle alveolar canal; maf, middle alveolar foramen; nrac1, rostral alveolar canal, first branch; rac2, rostral alveolar canal, second branch; rac3, rostral alveolar canal, third branch; rac4, rostral alveolar canal, fourth branch; rac5, rostral alveolar canal, fifth branch.

The infraorbital canal is the second main ramification and is the shortest among the three other canals. It opens on the lateral surface of the maxilla through a large foramen at the level of the second postcanine. Associated with the opening of the infraorbital canal there is a second foramen located dorsally to the infraorbital canal foramen (Figure 2). This foramen varies in diameter between specimens and both lateral sides of the rostrum in MCN‐PV 2264. In UFRGS‐PV‐596‐T, the secondary foramen is smaller compared with other specimens (MCN‐PV 2264 and UFRGS‐PV‐788‐T) (Figure 3a,b). This small foramen is associated with thin secondary projections that originate at the base of the branch of the rostral alveolar canal, and is located dorsal to the infraorbital canal. On the left side of the skull of UFRGS‐PV‐596‐T, the small foramen is closer to the infraorbital foramen, and both open together inside a small depression in the maxilla. In the right side of the skull, the small foramen is more distant from the infraorbital canal and the maxilla lacks a depression, similar to the other side. A similar condition is observed in specimen UFRGS‐PV‐788‐T, where a short branch projects from the base of the rostral alveolar canal, close to the branch between the infraorbital canal and the rostral alveolar canal (Figure 3e,f). The secondary branch has a larger diameter compared to the secondary branch of specimen UFRGS‐PV‐596‐T. In the holotype of *Riograndia* (MCN‐PV 2264), the small branch, or the foramen associated with the infraorbital canal, could not be identified (Figure 3c). Nonetheless, the left side has a secondary branch (Figure 3d), with a larger diameter compared to the secondary branch present in the specimen UFRGS‐PV‐596‐T. In the holotype (MCN‐PV 2264), the secondary branch projects at the level of the branching of the infraorbital canal with the rostral alveolar canal; however, it is positioned dorsally to the rostral alveolar canal. This secondary branch and foramina, present in the three specimens, have not been reported for other derived non‐mammaliaform prozostrodonts (e.g. *Brasilodon*, *Tritylodon*, and *Pachygenelus*; Benoit et al., 2020).

The most anterior branch is called the rostral alveolar canal (sensu Benoit et al., 2020) and has a diameter similar to the others. It bifurcates from the infraorbital canal at the level of the second postcanine (Figure 2). Despite being shorter than that in *Brasilodon* (Benoit et al., 2020), the rostral alveolar canal divides into at least three branches, similar to the condition reported for other prozostrodonts (Benoit et al., 2020; Kerber, Roese‐Miron, et al., 2024) (Figures 2 and 3). However, unlike *Brasilodon*, where the branches are together and restricted to the anterior end of the rostral alveolar canal (Benoit et al., 2020), in *Riograndia* the branches appear close to the separation point of the rostral alveolar canal and infraorbital canal and have different lengths and diameters. The first branch is dorsal and joins the pneumatization of the maxilla, and probably corresponds to the branch that supplies the canine alveolus observed by Benoit et al. (2020) in *Brasilodon*, identified as “internal terminations of the rostral alveolar canal.” In the three specimens of *Riograndia*, there is a clear connection between the maxillary canal and the pneumatization of the maxilla (Figures 2 and 3) and, in UFRGS‐PV‐596‐T, it is possible to observe that the pneumatization of the maxilla connects to the canine alveolus (see last paragraph of section 3.1). The second branch is shorter and located lateral to the first branch and ends in a foramen positioned dorsally to the infraorbital foramen, on the surface of the maxilla. This structure presents a variation in diameter among specimens, being smaller in UFRGS‐PV‐596‐T and larger in UFRGS‐PV‐788‐T and MCN‐PV 2264 (Figure 3). The third branch is the longest and extends laterally to the tooth row until ending in the rostral alveolar foramen, lateral to the canine root. This branch is different from the condition observed in *Brasilodon*, *Tritylodon*, and *Pachygenelus*, where the rostral alveolar foramina (or the external terminations of the rostral alveolar canal, in *Brasilodon*) are posterior to the root of the canine (Benoit et al., 2020). In addition to these branches, present in the three studied specimens and on both sides of the skull, some extra branches are observed in UFRGS‐PV‐788‐T and MCN‐PV 2264 (Figure 3). On the left side of the MCN‐PV 2264 and on the right side of the UFRGS‐PV‐788‐T, the rostral alveolar canal has more than three branches (Figure 3d,f). In MCN‐PV 2264, there is a branch ventral to the most anterior branch of the rostral alveolar canal that has a blind‐end (Figure 3d). In UFRGS‐PV‐788‐T, it is possible to observe two branches (Figure 3f). The first branch is more posterior and ventral to the most anterior branch of the rostral alveolar canal. It is in the same position as the secondary branch of the specimen MCN‐PV 2264, but it exits into a foramen. The second branch is shorter and lies dorsally to the most anterior branch of the rostral alveolar canal and also ends in a small foramen.

Two small branches are observed in the most posterior region of the maxillary canal, ending in two foramina at the contact between the maxilla and lacrimal. These branches are identified as zygomaticofacial canals (sensu Benoit et al., 2020). Nonetheless, Benoit et al. (2020) identified only one branch in all studied cynodonts, while in *Riograndia* (UFRGS‐PV‐596‐T), we identify two separate branches in the same region of similar diameter that end in two separate foramina (Figure 2). It is worth mentioning that, unlike *Brasilodon*, the zygomaticofacial canals of *Riograndia* do not contact the lacrimal canal, following the trend reported by Benoit et al. (2020).

**FIGURE 4:**
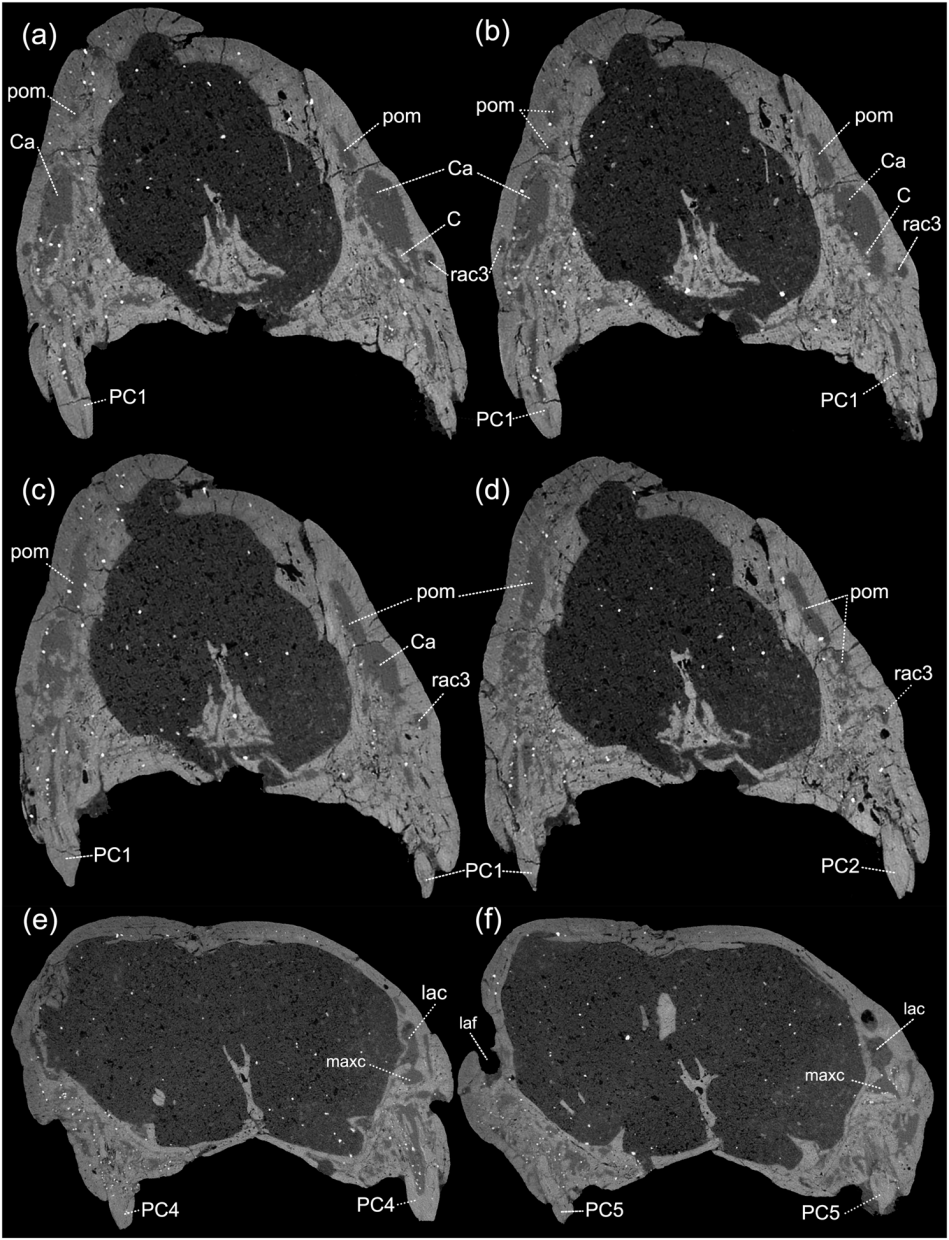
Digital sections of the posterior region of the upper canine of *Riograndia guaibensis* UFRGS‐PV‐596‐T, at the level of the first and second postcanines, being (a) the most anterior, (b, c) placed between (a, d), and (d) the most posterior. C, upper canine; Ca, upper canine alveolus; lac, lacrimal canal; laf, lacrimal foramen; maxc, maxillary canal; PC, upper postcanine; rac3, rostral alveolar canal, third branch; pom, pneumatization of the maxilla.

The pneumatization of the maxilla corresponds to a cavity located dorsally to the maxillary canal within the maxilla. It is connected to the maxillary canal through an opening at the level of the base of the rostral alveolar canal (Figure 2). This cavity is large and presents a series of trabeculae of larger diameter that cross this structure. The pneumatization of the maxilla begins at the level of the contact between the second and third postcanines, extends to the level of the canine root, and fuses with the alveolus of the canine (Figure 4). The pneumatization of the maxilla does not connect to any alveoli of the postcanine. Dorsally, the pneumatization of the maxilla extends across most of the height of the maxilla. On the lateral surface, two small foramina are observed deriving from the pneumatization of the maxilla (Figure 2). These foramina are aligned vertically and placed dorsal to the infraorbital foramen.

### 3.2 Phylogenetic analysis and distribution of characters of the maxillary canal

**FIGURE 5:**
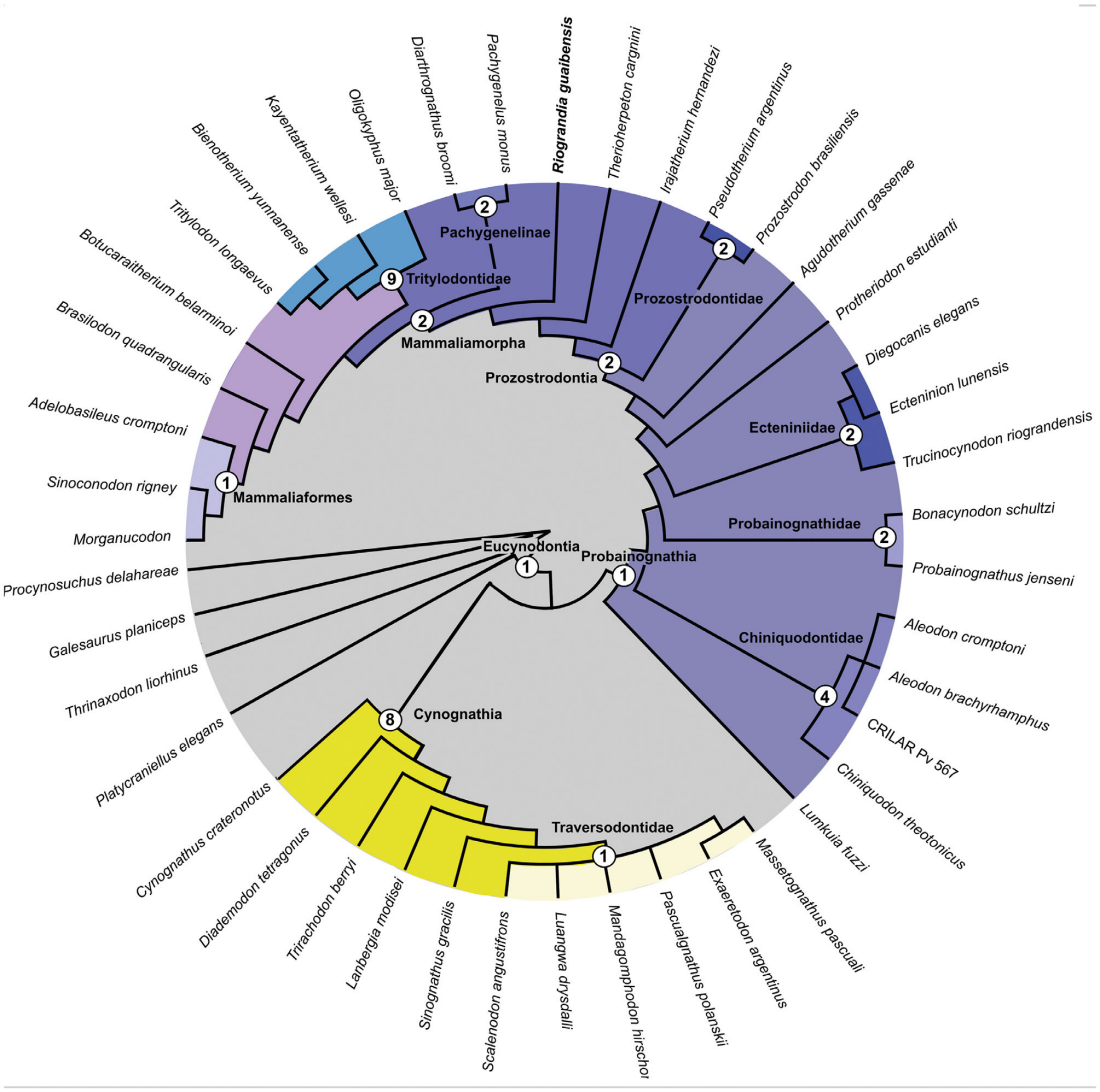
Strict consensus of the phylogenetic analysis of Eucynodontia. In yellow the group of Cynognathia and in blue scales the Probainognathia, including Mammaliaformes. Mammaliamorpha clade follows the definition presented by Abdala (2021). The numbers indicate the Bremmer support of each node.

The analysis recovered eight most parsimonious trees, with 506 steps (Consistency index: 0.457; Retention Index: 0.778). *Riograndia* is within Prozostrodontia and appears as the sister group to Pachygenelinae, Tritylodontidae, and the clade including *Botucaraitherium*, *Brasilodon*, and Mammaliaformes, which is supported by the character/state 97:0 → 1 (Figure 5). Using this approach of analysis (see Section 2), the internal relationships of Chiniquodontidae collapse in the strict consensus. Using the protocol Iter PCR (Pol & Escapa, 2009), *Riojanodon* and *Cromptodon* were identified as floating taxa and were removed from the strict consensus a posteriori (Figure 5).

Concerning the characters of the maxillary canal, of major interest here, the reduction or disappearance of the external nasal ramus (Ch. 150: 0 → 1) appears as a synapomorphy of Probainognathia (Figure 5). In probainognathians, the basal most taxon presents internal nasal and superior labial rami (Ch. 151: 0). They disappeared in *Probainognathus* (Ch. 151:1), but they are present in *Ecteninion* (Ch. 151:0). In Prozostrodontia, it is absent (Ch. 151:1) (Figure 5). The lacrimal canal and zygomaticofacial canal are fused (Ch. 152:0) in most cynodonts and separated (152:1) in *Probainognathus* (based on cf. *Probainognathus*, see comments in Fernández et al., 2011) plus prozostrodonts (except for *Brasilodon* in which both canals become fused, Ch. 152: 0) (Figure 5). The maxillary recess (=antrum) and maxillary canal are separated (Ch. 153: 1) in all prozostrodonts in which such structures are known. Finally, the presence of an anteroposteriorly elongated maxillary antrum is present in cynognathian gomphodonts (Ch. 154:1).

## 4 DISCUSSION

**FIGURE 6:**
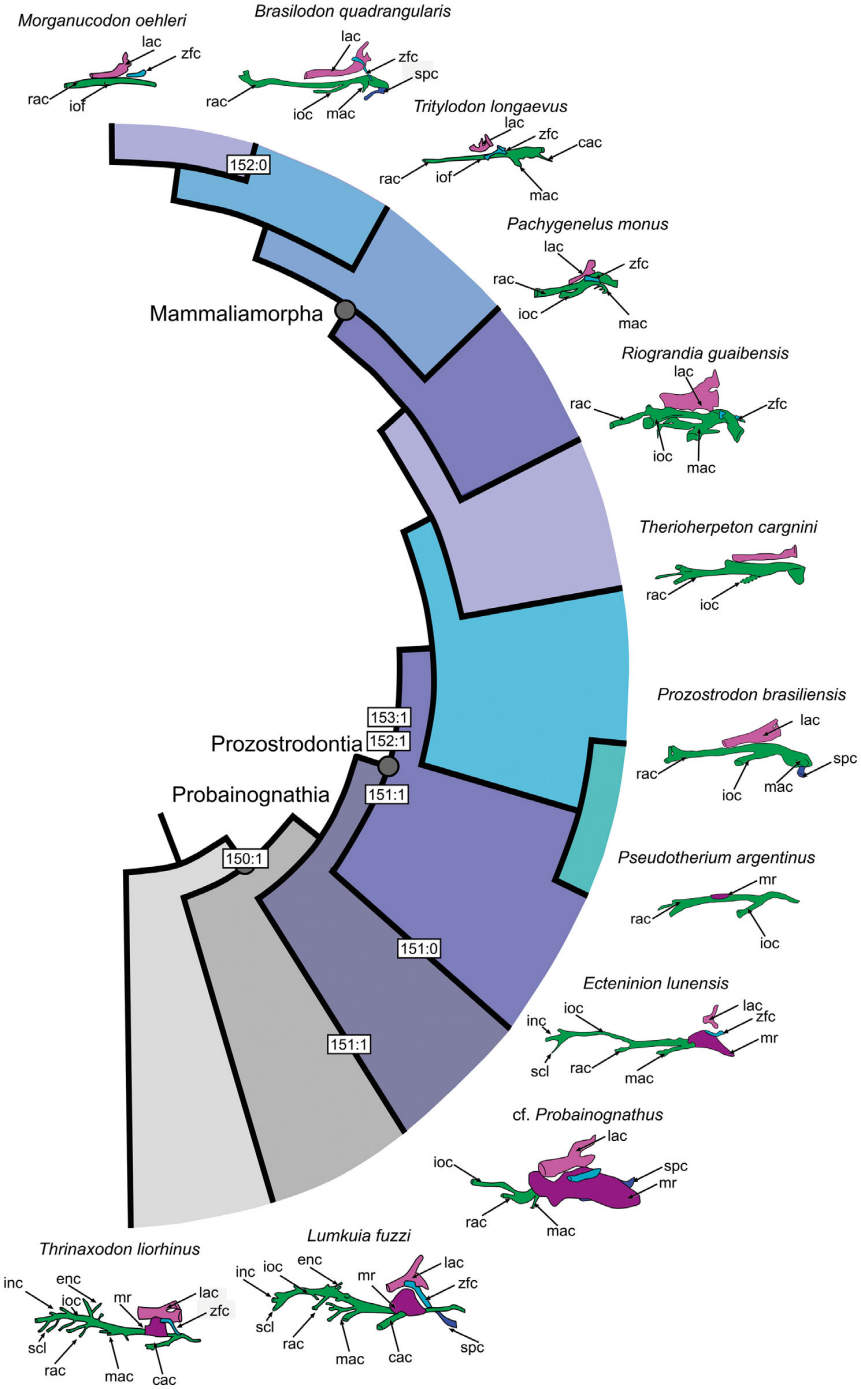
Evolution of the maxillary canal in Probainognathia according to the framework proposed by Benoit et al. (2020). The numbers indicate the character/state changes (see main text). Sources: *Pseudotherium* (after Wallace et al., 2019), *Prozostrodon*, *Therioherpeton* (after Kerber, Roese‐Miron, et al., 2024), *Riograndia* (this work), and other cynodonts from Benoit et al. (2020). Structures identified in *Pseudotherium*, *Prozostrodon*, and *Therioherpeton* are identified following the pattern of prozostrodonts by Benoit et al. (2020), especially *Brasilodon*. Cac, caudal alveolar foramen; enc, external nasal canal; inc, internal nasal canal; ioc, infraorbital canal; iof, infraorbital foramen; lac, lacrimal canal; mac, middle alveolar canal; mr, maxillary recess; rac, rostral alveolar canal; raf, rostral alveolar foramen; slc, superior labial canal.

Probainognathia underwent significant changes in the configuration of the innervation of the maxillary nerve, and consequently in the morphology of the maxillary canal (Benoit, Fernandez, et al., 2016; Benoit, Manger, et al., 2016; Benoit et al., 2020). The earliest probainognathian (i.e. *Lumkuia*) still retained a plesiomorphic pattern shared with other non‐probainognathian cynodonts (Figure 6). In *Probainognathus* (cf. *Probainognathus*; see comments in Fernández et al., 2011), the disappearance of the superior, internal, and external labial canals is observed (Figure 6). From this point onwards, these branches are no longer within the maxilla but within the soft tissues overlaying it. Thus, *Probainognathus* is the earliest‐diverging probainognathian candidate to possess vibrissae (Benoit et al., 2020). With the emergence of Prozostrodontia during the Carnian, the canal undergoes further modifications. Now, the rostral alveolar canal (always oriented towards the base of the canine) becomes longer than the infraorbital portion of the maxillary canal (Figure 6).

In general, the anatomy of the branches of the maxillary canals of *Riograndia* presents the expected pattern for non‐mammaliaform prozostrodonts (sensu Benoit et al., 2020). It presents three main branches, namely the middle alveolar canal, infraorbital canal, and rostral alveolar canal. This condition is consistent with the phylogenetic placement of *Riograndia* within Prozostrodontia (e.g. Kerber, Roese‐Miron, et al., 2024; Liu & Olsen, 2010; Martinelli et al., 2024; Stefanello et al., 2023). Furthermore, Benoit et al. (2020) observed that *Brasilodon* and *Tritylodon* present an intermediate condition between the early diverging cynodonts, where the rostral alveolar canal supplied the external surface of the maxillary bone, and the more derived condition where the rostral alveolar canal supplied the canine alveolus, as in *Pachygenelus* and *Morganucodon*. *Riograndia* shares the same intermediate condition, with the rostral alveolar canal presenting foramina on the external surface of the maxilla, as well as having a connection with the canine alveolus (Figure 2).

However, we observed variations in the morphology of some of the structures in *Riograndia*. They include the separation of the maxillary canal from the lacrimal canal, the position of the middle alveolar canal in comparison to other cynodonts, the anatomy of the rostral alveolar canal, and the presence of pneumatization near the posterior portion of the canine alveolus, connected to the maxillary canal.

The first difference present in *Riograndia* is the separation of the maxillary canal from the lacrimal canal. According to Benoit et al. (2020), the maxillary canal in Prozostrodontia is completely separated from the lacrimal canal. Nonetheless, we consider that the separation of the maxillary canal from the lacrimal canal is incomplete in *Riograndia*, since there are two points of contact between canals along their length (see Description). These points of contact between the maxillary and lacrimal canals are reduced compared to what is observed in *Thrinaxodon* (Benoit et al., 2020; Fonseca et al., 2024). In our interpretation based on three individuals of *Riograndia*, we clearly observe that the lacrimal canal and the maxillary canal presents points of contact through non‐ossified areas indicating that the complete separation of the maxillary canal is not present in all Prozostrodontia, and this feature occurs in more derived forms, such as *Brasilodon* (sensu Benoit et al., 2020) or Mammaliaformes. Despite this, the separation of the maxillary canal from the lacrimal canal is well‐advanced in this taxon, and they seem to be independent of each other.

The opening position of the middle alveolar canal in *Riograndia* is different from that described for some prozostrodonts (Benoit et al., 2020; Kerber, Roese‐Miron, et al., 2024). While in most taxa in which the morphology of this canal is known, the opening of the middle alveolar canal is ventrally facing, whereas in *Riograndia* the opening faces laterally, at the base of the zygomatic arch. The change in the position of the opening is presumably related to the shortening of the snout in *Riograndia* and the enlargement of the base of the zygomatic arch. This condition was not observed in any other Prozostrodontia and it is probably related to anatomical adaptations for a specialized life habit.

**FIGURE 7:**
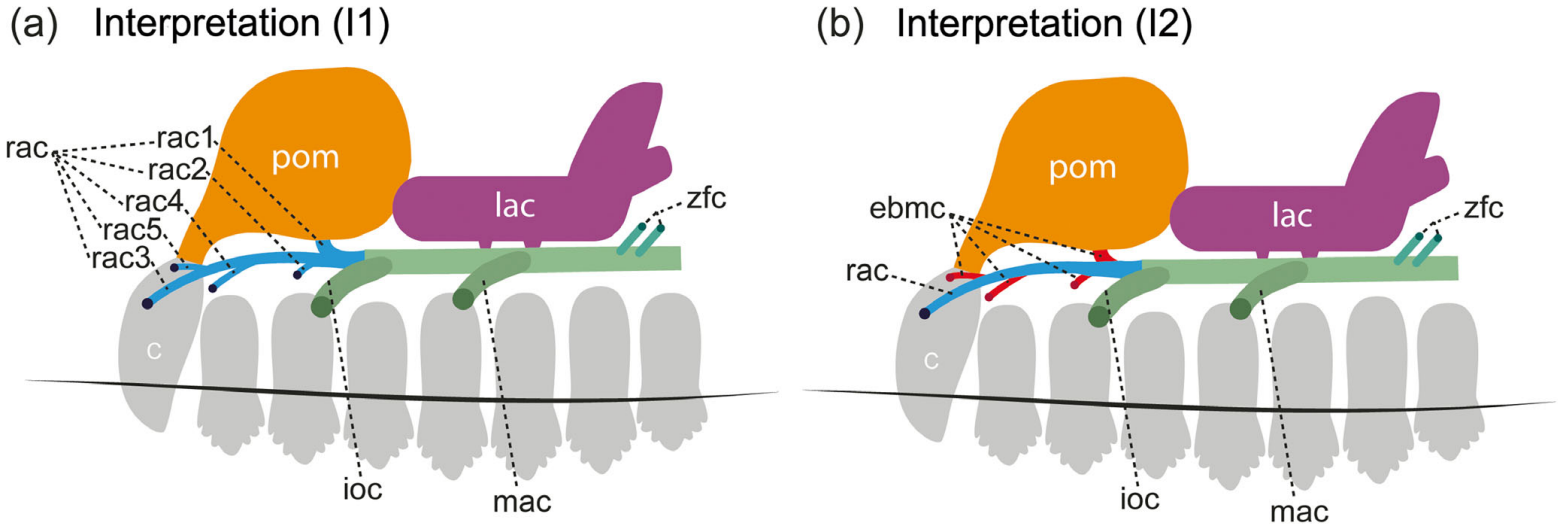
Scheme illustrating the two interpretations of the anatomy (a, b) discussed in the text about the identification of the rostral alveolar canal (rac) in *Riograndia*, based on all studied specimens. C, upper canine; ebmc, extra branches of the rostral alveolar canal; ioc, infraorbital canal; lac, lacrimal canal; laf, lacrimal foramen; mac, middle alveolar canal; pom, pneumatization of the maxilla; rac, rostral alveolar canal; rac1, rostral alveolar canal, first branch; rac2, rostral alveolar canal, second branch; rac3, rostral alveolar canal, third branch; rac4, rostral alveolar canal, fourth branch; rac5, rostral alveolar canal, fifth branch; zfc, zygomaticofacial canal.

One of the traits present in *Riograndia* that stands out most is the morphology of the rostral alveolar canal. It is different from that hitherto known for non‐mammaliafom cynodonts and allows two possible interpretations regarding its identification (Figure 7). The first interpretations (I1) considers that the rostral alveolar canal of *Riograndia* is shorter than in *Brasilodon* and other prozostrodontians, with its first branches close to the separation of the infraorbital canal, being formed by three main branches, and may have extra branches (see Figures 3 and 7a). In the alternative interpretation (I2), *Riograndia* presents the three main branches of the maxillary canal, where the third branch of the rostral alveolar canal (rac3—proposed in this paper) would comprise the branch of the rostral alveolar canal as well as the “rac” identified by Benoit et al. (2020), with other extra, randomly formed branches (in interpretation 1 those branches are considered as rac1, rac2 in UFRGS‐PV‐596‐T, plus rac4 and rac5 in UFRGS‐PV‐788‐T and MCN‐PV 2264).

In our first interpretation, the rostral alveolar canal of *Riograndia* presents a pattern similar to that described for *Brasilodon*, *Therioherpeton*, and *Prozostrodon* with the rostral alveolar canal divided in branches (Benoit et al., 2020; Bonaparte & Barberena, 2001; Kerber, Roese‐Miron, et al., 2024). However, unlike *Brasilodon* (Benoit et al., 2020) in which the rostral alveolar canal divides at the anterior end into branches of small length and diameter, the rostral alveolar canal in *Riograndia* branches immediately after separating from the infraorbital canal, in at least three main branches plus the accessory branches present in some individuals (UFRGS‐PV‐788‐T and MCN‐PV 2264) that have different diameters and length (see Description). The branches are easily mapped and the presence of a branch that connects to the canine socket (through the pneumatization of the maxilla), would corroborate our first interpretation (Figure 7), that is the one used in the description. According to Benoit, Fernandez, et al. (2016) and Benoit et al. (2020), in specimens in which the canine is present, the presence of a branch that is oriented towards the base of the canine root would confirm the identification of the rostral alveolar canal. We believe the nerve (and probably vessels) pass through the pneumatization of the maxilla and reach the base of the canine alveolus once the pneumatization of the maxilla connects the branch of the rostral alveolar canal and the canine alveolus, and it has smaller foramina that contact the external surface of the jaw bone (see Description). Furthermore, this branch that connects to the root of the canine is the first (most posterior) branch to separate from the other branches of the rostral alveolar canal (Figure 3) in the three specimens of *Riograndia*. The presence of this branch at the beginning of the separation of the rostral alveolar canal allows us to confirm the identification of this branch as being the rostral alveolar canal. Therefore, the first interpretation (I1) seems more plausible to us regarding the identification of the rostral alveolar canal (Figure 7).

Alternatively, in our second interpretation is possible that the arrangement of the foramina on the surface of the maxilla of *Riograndia* (Figure 1 and 7b) corresponds to the pattern of three main foramina as observed in *Brasilodon*, *Tritylodon*, *Therioherpeton*, and *Prozostrodon* (the most anterior being subdivided into three smaller foramina in *Brasilodon*, *Therioherpeton*, and *Prozostrodon*) (Benoit et al., 2020; Kerber, Roese‐Miron, et al., 2024). In this hypothesis, the most anterior foramen corresponds to the exit of the rostral alveolar canal, which would be more elongated than the others and would end close to the root of the canine in *Riograndia*, as described by Benoit et al. (2020) for other taxa. The remaining branches would comprise extra branches (ebmc) identified as rac1, rac2, rac4, and rac5 in I1 (Figure 7). However, we identified that the rac1, rac2, and rac3 branches are present in all three specimens analyzed of *Riograndia* and present a similar morphological pattern. The other branches (rac4 and rac5) are variable according to the specimens and may not even present an exit foramen (rac4 of the MCN‐PV 2264—Figure 3d). Another difference observed in *Riograndia* is the most anterior branch (which would correspond to the rostral alveolar canal of Benoit et al., 2020) ends laterally at the root of the canine in *Riograndia* and not posteriorly to the canine as observed in *Brasilodon*, *Tritylodon*, *Therioherpeton*, and *Prozostrodon* (Benoit et al., 2020; Kerber, Roese‐Miron, et al., 2024). Based on both interpretations, we follow the first (I1) as the most reasonable one. Despite these differences, we were able to identify the three main branches (middle alveolar canal, infraorbital canal, and rostral alveolar canal; sensu Benoit et al., 2020) present in *Riograndia* and, the presence of these extra branches could be a unique condition of *Riograndia*, related to the shortening of the snout.

Another different feature in *Riograndia* is the extension of the third branch of the rostral alveolar canal. Differing from *Brasilodon* and other Prozostrodontia, where the branches of the rostral alveolar canal end in foramina posterior to the canine (Benoit et al., 2020), in *Riograndia* the third branch of the rostral alveolar canal projects anteriorly, ending in a foramen positioned lateral to the root canine root. This repositioning of the third branch of the rostral alveolar canal may be associated with the shortening of the snout of *Riograndia*. Other features inside the nasal cavity related to the shortening of the snout were reported by Fonseca et al. (2024), such as reduction of the participation of the maxilla on the floor of the nasal cavity and the shortening of the vomer. Furthermore, the third branch of the rostral alveolar canal has a slightly smaller diameter than the other branches of the maxillary canal (middle alveolar canal and infraorbital canal) and the foramen where the branch ends resemble the topology of other foramina found on the surface of the maxilla (Video 1). In addition to the position of the third branch of the rostral alveolar canal in *Riograndia*, we observed that this branch may have extra branches along its length which can vary in number, and may only appear on one side of the skull (Figure 3). This extra foramen has not been reported in any Prozostrodontia taxon so far (e.g. Benoit et al., 2020) and presents variation in its diameter, being reduced in UFRGS‐PV‐596‐T and larger in UFRGS‐PV‐788‐T and MCN‐PV 2264. Furthermore, the position of the foramina may vary along the surface of the bone among specimens (UFRGS‐PV‐596‐T, UFRGS‐PV‐788‐T, and MCN‐PV 2264) and between the two sides of the skull (e.g. UFRGS‐PV‐596‐T) (Figure 3). These variations are expected for smaller branches of nerves and vessels.

**VIDEO 1:** 3D reconstruction of the rostral region of *Riograndia* UFRGS‐PV‐596‐T and maxillary canals and other associated structures.

Finally, the pneumatization recognized inside the facial process of the maxilla of *Riograndia* has not been previously described for any other non‐mammaliaform cynodonts. This structure, marked in orange in the 3D reconstructions (Figures 2 and 3), is not related to the maxillary pneumatization or maxillary recess recognized in many non‐mammaliaform cynodonts or early mammaliaforms (e.g. Crompton et al., 2017; Franco et al., 2021; Kermack et al., 1981). Along the evolutionary history of synapsids, sinuses related to the maxillary bone have been mentioned in several taxa, with still limited information regarding their functionality and homology within the clade. Sigurdsen (2006) describes a maxillary sinus close to the root of the canine (i.e. anterior maxillary sinus) in akidnognathid therocephalians from the late Permian of the Karoo Basin (South Africa). Lillegraven and Krusat (1991) describe in *Haldanodon exspectatus* a cavity inside the maxillary bone, with the same characteristics as the pneumatization of the maxilla of the *Riograndia*, presenting associated foramina on the surface of the maxilla, and identifying it as a sinus. A similar structure is described for the multituberculate *Nemegtbaatar gobiensi* (Hurum, 1994) and identified as a maxillary sinus. However, in comparison to *Riograndia* and *Haldanodon*, the maxillary sinus of *Nemegtbaatar* appears to be larger and occupies the entire length of the maxilla. In *Riograndia*, this structure runs most of the height of the maxilla, and connects the branch of the rostral alveolar canal with the most dorsal region of the upper canine alveolus (see Description).

The pneumatization of the maxilla appears to be formed by the reabsorption of bone subsequent to the formation of the maxillary bone, thus forming an irregular structure similar to the structures identified in the skulls of different groups of current reptiles (e.g. Fonseca et al., 2020; Rayfield & Milner, 2008; Witmer, 1995, 1997; Witmer & Ridgely, 2008, 2009) and current mammals (e.g. Curtis and van Valkenburgh, 2014; Curtis et al., 2015; Kielan‐Jaworowska et al., 1986; Koppe et al., 1999; Rossie, 2006; Smith et al., 2005). The process of formation of this pneumatization is linked to the reduction of bone mass in certain areas of the skull and is influenced by the development of different areas of the skull, such as the development of the orbit and dentition (Smith et al., 2005; Witmer, 1997). Although it is difficult to confirm, we believe that the structure identified here as a pneumatization of the maxilla is related to structures identified as sinus in *Haldanodon* (Lillegraven & Krusat, 1991) and the maxillary sinus of *Nemegtbaatar* (Hurum, 1994). The position of this structure inside of the maxillary bone in *Haldanodon* and *Nemegtbaatar*, as well as the presence of foramina related to this cavity on the lateral surface of the former taxon, could corroborate this relationship. It is important to note that the structure called here sinus of the maxilla is homologous with the sinus and maxillary sinus pointed out by Lillegraven and Krusat (1991) and Hurum (1994), respectively. Nonetheless, it is different from the structure known as a maxillary sinus (or recess), located in the posteroventral region of the nasal cavity, formed mainly by the palatine, and identified in many non‐mammaliaform cynodonts and early mammaliaforms (e.g. Benoit et al., 2020; Crompton et al., 2017; Davis et al., 2022; Fonseca et al., 2024; Kermack et al., 1981; Martinelli et al., 2024). Furthermore, we are unable to confirm whether there is a homology between the sinus of the maxilla, present in the previously mentioned taxa, with the maxillary sinus described for current mammals (e.g. Smith et al., 2005), even though it presents a similar topology and morphology.

Better understanding the development of the maxillary sinus in current mammals could help in the sinus of the maxilla in non‐mammaliaform cynodonts. The maxilla sinus in extant mammals occurs through the reabsorption of bone mass within the maxilla during ontogenetic development, and the subsequent opening of the wall that divides the sinus of the maxilla from the nasal cavity (Smith et al., 2005). This could indicate that in non‐mammaliaform cynodonts (e.g. *Riograndia*), early mammaliaforms (e.g. *Haldanodon*) and multituberculates (e.g. *Nemegtbaatar*), this structure is formed throughout the development of the skull and would remain isolated from the nasal cavity even at more advanced ontogenetic stages.

We hypothesize that the branch of the rostral alveolar canal that passes through the sinus of the maxilla is the internal termination of the rostral alveolar canal described by Benoit et al. (2020). However, although Benoit et al. (2020) did not identify this structure, we believe that the internal termination of the rostral alveolar canal branch of *Brasilodon* may connect to the canine alveolus through a pneumatized region, as in *Riograndia*. Furthermore, we observe that small foramina branch from this structure and exit on the lateral surface of the maxillary bone, indicating that the nerve branch and/or blood vessels branch inside the maxillary sinus and project to the surface of the snout.

## 5 CONCLUSION

*Riograndia* presents a morphological pattern similar to that of the other Prozostrodontia, where the maxillary canal is almost completely separated from the lacrimal canal, and there are three main branches, which run along the lateral region of the snout. The evidence provided by three specimens of *Riograndia* confirms that the separation of the lacrimal canal from the maxillary canal presents points of contact through non‐ossified areas, demonstrating that the separation process is therefore still incomplete in this taxon and not a preservational artifact. Furthermore, we observed that the anatomy of the rostral alveolar canal of *Riograndia* presents a different pattern from that previously described in the literature, with a variety in the number of extra branches which end in foramina on the lateral surface of the maxilla, in addition to presenting variation among individuals and between both sides of the skull of the same individual. Variation between individuals and between sides of the skull is something expected in a population, and also throughout ontogeny. Therefore, the small variations in the morphology of this taxon demonstrate that the interpretation of characters related to internal structures, such as foramina, must be treated cautiously and with techniques such a tomographic imaging that access the interior of the bone/skull. This allows mapping the ramifications of the structures, making it possible to identify homologous features.

In addition to the anatomical variations of the maxillary canal, the presence of pneumatization in the maxilla of *Riograndia* is interesting, since this structure is directly associated with the distribution of some branches of the maxillary canal. Furthermore, this structure resembles areas of pneumatization of the skull present in other groups, such as extant and fossil reptiles (e.g. maxillary sinus in living crocodilians and dinosaurs) and extant mammals (e.g. frontal sinus). However, it is not possible to know whether this is the same structure or even its functionality. We suggest that this structure may be present in other non‐mammaliaform cynodonts and may be important to understanding of the biology and phylogeny of the group in future works.

## AUTHOR CONTRIBUTIONS

**Pedro Henrique Morais Fonseca:** Conceptualization; writing – original draft; investigation; writing – review and editing; methodology; data curation; project administration. **Agustín Guillermo Martinelli:** Conceptualization; investigation; writing – original draft; writing – review and editing; methodology; supervision; data curation; project administration. **Pamela G. Gill:** Data curation; investigation; writing – review and editing. **Emily J. Rayfield:** Investigation; writing – review and editing; data curation; project administration. **Cesar Leandro Schultz:** Writing – review and editing; data curation; investigation; project administration. **Leonardo Kerber:** Investigation; writing – review and editing; data curation. **Ana Maria Ribeiro:** Investigation; writing – review and editing; data curation. **Marina Bento Soares:** Conceptualization; writing – review and editing; supervision; data curation; project administration.

## CONFLICT OF INTEREST STATEMENT

The authors declare no competing financial interests.

## Supporting information


Data S1.

Data S2.

## Data Availability

CT‐Scans and STL data are available on request from the authors.
